# Imputation accuracy of wheat genotyping-by-sequencing (GBS) data using barley and wheat genome references

**DOI:** 10.1371/journal.pone.0208614

**Published:** 2019-01-07

**Authors:** Hadi Alipour, Guihua Bai, Guorong Zhang, Mohammad Reza Bihamta, Valiollah Mohammadi, Seyed Ali Peyghambari

**Affiliations:** 1 Department of Agronomy, Kansas State University, Manhattan, Kansas, United States of America; 2 Department of Plant Breeding and Biotechnology, Faculty of Agriculture, Urmia University, Urmia, Iran; 3 USDA-ARS, Hard Winter Wheat Genetics Research Unit, Manhattan, Kansas, United States of America; 4 Department of Agronomy and Plant Breeding, Faculty of Agriculture, University of Tehran, Karaj, Iran; Ben-Gurion University, ISRAEL

## Abstract

Genotyping-by-sequencing (GBS) provides high SNP coverage and has recently emerged as a popular technology for genetic and breeding applications in bread wheat (*Triticum aestivum* L.) and many other plant species. Although GBS can discover millions of SNPs, a high rate of missing data is a major concern for many applications. Accurate imputation of those missing data can significantly improve the utility of GBS data. This study compared imputation accuracies among four genome references including three wheat references (Chinese Spring survey sequence, W7984, and IWGSC RefSeq v1.0) and one barley reference genome by comparing imputed data derived from low-depth sequencing to actual data from high-depth sequencing. After imputation, the average number of imputed data points was the highest in the B genome (~48.99%). The D genome had the lowest imputed data points (~15.02%) but the highest imputation accuracy. Among the four reference genomes, IWGSC RefSeq v1.0 reference provided the most imputed data points, but the lowest imputation accuracy for the SNPs with < 10% minor allele frequency (MAF). The W7984 reference, however, provided the highest imputation accuracy for the SNPs with < 10% MAF.

## Introduction

Wheat (*Triticum aestivum* L.) is a major staple food crop in the world. The fast-growing world population demands wheat production to be increased up to 70% by 2050 to feed estimated world population of approximately nine billion [1–3]. Application of advanced genomic technologies in breeding can speed up genetic improvement of new wheat varieties to meet the challenge [4]. Next-generation-sequencing (NGS) technologies have revolutionized throughput and greatly reduced DNA sequencing cost, which makes it feasible for routine screening of breeding materials [5]. Genotyping-by-sequencing (GBS) is one application that sequences a subset of a complex genome and also multiplexes various numbers of samples to lower the genotyping cost [6]. GBS can discover and genotype single nucleotide polymorphisms (SNPs) simultaneously and it is a valuable platform for crop breeding and genomic research [6].

SNPs are the most abundant type of sequence variations in plant genomes [7] and therefore suitable for studies that require a large number of markers to be assayed such as marker-trait association analysis, genetic map construction, quantitative trait locus (QTL) screening, genomic selection, and analysis of population structure and genetic variation [8]. High-throughput SNP genotyping platforms have been successfully used for diploid crops such as maize [9] and barley [10]. Wheat, however, is polyploid and has a huge genome (~17 Gb) with abundant repetitive DNA (> 80%), which present major challenges to direct sequencing the genome for developing high-density SNP maps [11]. Recently, a GBS protocol [5] has been optimized for cereal crops including wheat and can generate thousands of SNPs with reasonably low cost [12–14]. However, abundance of missing data due to low sequencing coverage significantly reduces number of usable SNPs and lowers marker density [5]. High marker density will improve accuracy of many downstream analyses such as QTL mapping, genome-wide association studies (GWAS) and genomic selection [15, 16]. Sequencing depth and library complexity and quality may all affect number of missing data. Increase in sequencing depth can lower missing data rate, but also increase sequencing cost. Marker imputation using available information from reference genomes can increase usable SNPs without increasing sequencing cost. Several imputation algorithms including IMPUTE [17], MaCH [18], fastPHASE [19], BEAGLE [20] have been developed to assign allelic status of missing values to genotypic data. Among those algorithms, IMPUTE and MaCH use hidden Markov model (HMM) and Markov chain Monte Carlo (MCMC) iterations to conduct subsampling, and the haplotypes in each iteration are considered as a sample from the haplotype pool. FastPHASE and BEAGLE, however, cluster haplotypes and collapsed total number of haplotypes into a smaller number of “ancestral” haplotypes [21]. Although both BEAGLE and fastPHASE use a hidden Markov model, BEAGLE is more parsimonious by allowing fewer possible transitions and emissions. In addition, fastPHASE fixes the number of clusters in the model, whereas BEAGLE allows dynamic change of number of clusters to fit localized linkage disequilibrium (LD) patterns [22]. Therefore, BEAGLE has been used to impute missing data in many studies [23–25]. Length of LD blocks greatly affects imputation accuracy because recombination breaks allelic associations. The markers that are common between samples and a reference panel serve as anchors to guide genotype imputation of any missing haplotypes within an LD block [26].

Imputation strategies may vary from species to species depending on availability of reference genomes and well-saturated reference linkage maps in a species. A more complete reference genome allows proper alignment and ordering of the sequenced tags and helps impute low coverage data [27]. To date, three wheat reference genomes and one barley reference genome have been reported [28]. Among the three wheat reference genomes, Chinese Spring survey sequence (CSSS) [29] has 10.2 Gb of sequences generated from Illumina NGS and W7984 reference has 9.1 Gb of sequences that were assembled using large-insert libraries and the three homoeologous genomes were assembled separately [30]. W7984 reference has lower genome coverage than CSSS, but higher assembly quality. Wheat IWGSC RefSeq v1.0 reference is the newest version of wheat reference genome with the best assembly quality, contains 14.5 Gb sequences with 94% genome coverage and was assembled using POPSEQ data and HiC map (chromosome conformation capture) [https://wheat-urgi.versailles.inra.fr/Seq-Repository/Assemblies]. Here we used the four reference genomes to compare imputation efficiencies and accuracies of wheat GBS data.

## Materials and methods

### Plant materials

A total of 384 accessions of Iranian wheat accessions [http://biogeo.ucdavis.edu/projects/iranwheat] were kindly provided by the United States Department of Agriculture (USDA) germplasm collection (https://npgsweb.ars-grin.gov/gringlobal/search.aspx), International Center for the Improvement of Maize and Wheat (CIMMYT), University of Tehran (UT), and Seed and Plant Improvement Institute (SPII), Karaj, Iran [31]. The wheat collection includes 276 Iranian landraces collected from different climates between 1937 and 1968 and 108 cultivars released in Iran between 1942 and 2014. Genomic DNA of the accessions were extracted from two-week-old seedling leaves using a modified cetyltrimethyl ammonium bromide (CTAB) method [32]. DNA concentration was quantified using the Quant-iT PicoGreen dsDNA Assay (Life Technologies Inc., NY) and normalized to 20 ng/μl.

### GBS library preparation and sequencing

The GBS library was constructed following Poland et al. [13]. In brief, genomic DNA of each sample was double-digested with *PstI* (CTGCAG) and *MspI* (CCGG) restriction enzymes (New England BioLabs Inc., Ipswich, MA, USA), and ligated to barcoded adapters using T4 ligase (New England BioLabs Inc.). All the ligated products were pooled and cleaned up using the QIAquick PCR Purification Kit (Qiagen Inc., Valencia, CA, USA). Primers complementary to both adaptors were used for PCR. PCR amplification started at 95 °C for 5 min, followed by 16 cycles of 95 °C for 30 s, 62 °C for 20 s and 68 °C for 1 min and ended by a final extension step at 72 °C for 5 min. The PCR product was then cleaned up again using the QIAquick PCR Purification Kit, and quantified using Bioanalyzer 7500 Agilent DNA Chips (Agilent Technologies, Inc.). After size-selection for 250–300 bp fragments in an E-gel system (Life Technologies Inc.), concentration of the library was evaluated using a Qubit 2.0 fluorometer and Qubit dsDNA HS Assay Kit (Life Technologies Inc.). The size-selected library was sequenced on an Ion Proton system (Life Technologies Inc.).

Sequence reads were first trimmed to 64 bp, and identical reads were grouped into sequence tags. Unique sequence tags were aligned internally to identify SNPs within the tags allowing mismatches of up to 3 bp. SNPs were called using the Universal Network Enabled Analysis Kit (UNEAK) GBS pipeline [33] in TASSEL 3.0 bioinformatics analysis package [34]. Tags with low quality score (< 15) were removed. SNPs with heterozygotes or a minor allele frequency > 10% were discarded to reduce the false positive markers. Only SNPs with lower than 80% missing data were used for this study. BLASTn analysis was carried out to align sequence tags to the four genome references including one from the barley reference genome [28], and three from wheat reference genomes, the flow-sorted Chinese Spring survey sequence (CSSS) [29], the Popseq W7984 sequence reference [30] and IWGSC RefSeq v1.0 [https://wheat-urgi.versailles.inra.fr/Seq-Repository/Assemblies]. The purpose of using the barley reference genome is to show efficiency of using reference genomes of closely related species to impute missing data in cases where reference genome sequence is absent in some species. If a SNP could be mapped in multiple chromosome positions, the position with the lowest E-value was used to represent the SNP location.

In this study, imputation was performed using BEAGLE v3.3.2 [20] and the four genome reference genomes. BEAGLE used a phasing algorithm to determine haplotype phase for each individual and to impute the missing values based upon allele frequencies. This was done by constructing local haplotype clusters and then sampling a number of haplotypes for each individual from a special class of HMM. Probability of each possible haplotype was estimated using the genotypic information and a forward-backward algorithm [35]. Then, new haplotypes for the individuals were sampled according to the conditional probabilities to reconstruct the local haplotype cluster as input for next iteration. This process was repeated several times. To achieve a high level of phasing accuracy in the end, the most-likely haplotypes for each individuals were imputed using the Viterbi algorithm [35].

Imputation accuracy was calculated by comparing the imputed SNPs after five, six and seven sequencing runs to the actual SNPs called from eight sequencing runs. Two files including one file of the actual SNP data from five (359 million reads), six (421 million reads), seven (488 million reads) and eight (566 million reads) sequencing runs, and an imputed data file generated from the five, six and seven sequencing runs were compared to calculate the number of correctly imputed data points. The ratio between correctly imputed and total imputed data points was used to estimate imputation accuracy [36]. To evaluate the relationship between imputation accuracy and allele frequency, allele frequencies from the original data file were calculated for each SNP.

## Results

**Fig 1:**
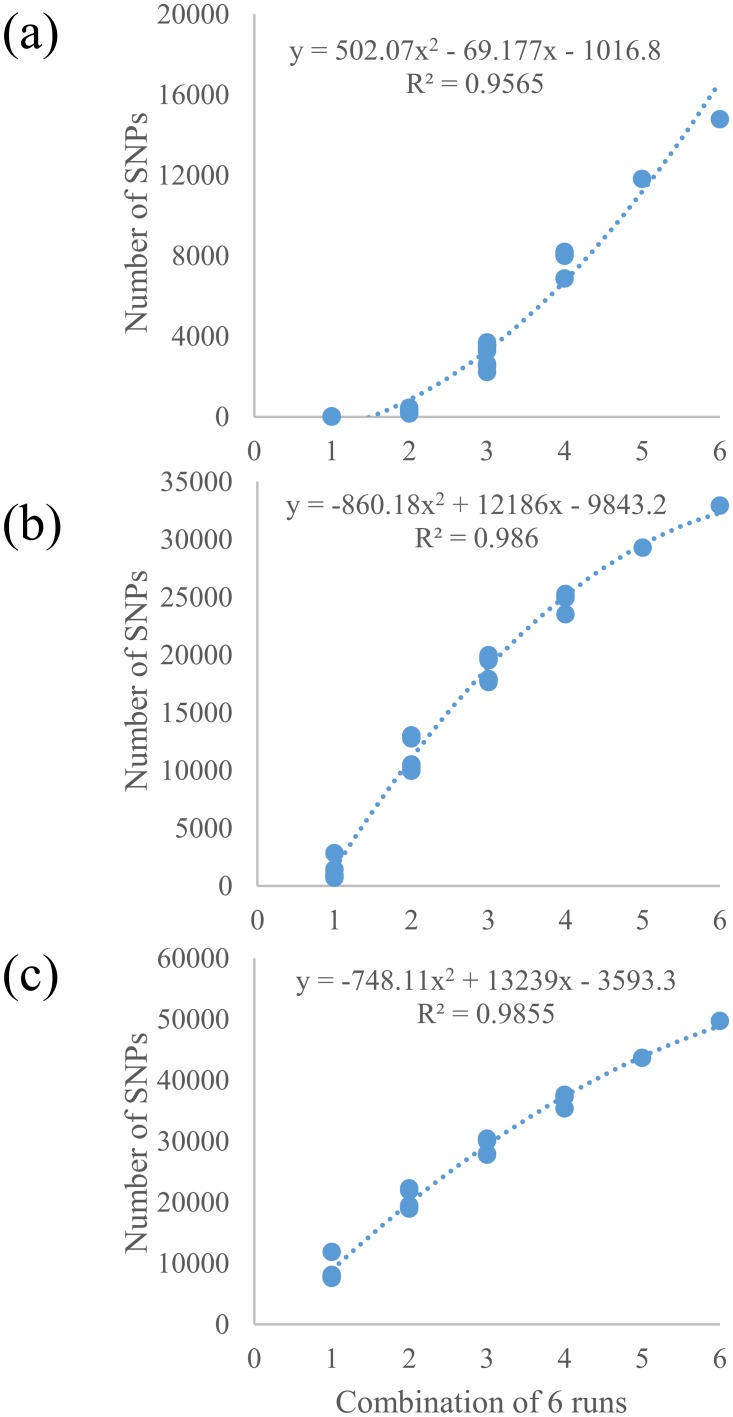
Relationship between numbers of sequencing runs and the numbers of SNPs with (a) <20%, (b) <50%, and (c) <80% missing data for the first library of 288 Iranian wheat genotypes.

Two GBS libraries were generated for the 384 wheat accessions with 276 landraces and 12 cultivars in library 1 and 96 cultivars in library 2. To minimize missing data, an average of two sequencing runs was performed for each plate of 96 samples, therefore, library 1 with three plates of samples was run a total of six times and library 2 with one plate of samples was run twice. Eight sequencing runs generated a total of 566,439,207 reads from the two libraries with 81% (458,363,607) of high-quality barcoded reads, from which 133,039 unique SNPs were identified including 16,506, 38,642 and 65,560 SNPs with <20%, <50% and <80% missing data, respectively. To determine the relationship between number of GBS-SNPs and number of sequencing runs, numbers of SNPs were calculated for each increased run from first to six sequencing runs of the library 1 (Fig 1). The number of SNPs with <20% missing data was concave up as the run number increased (Fig 1a) but concave down for the numbers of SNPs with <50% and <80% missing data (Fig 1b and 1c).

The average SNP density was 3.87 SNPs per Mbp when the SNPs with <80% missing data were counted (Table 1). The sequence tags containing the SNPs with <80% missing data were used to blast against each of the four references to map those SNPs to unique chromosome locations. The barley reference genome mapped the lowest percentage of sequencing tags (23.14%, Table 1), whereas IWGSC RefSeq v1.0 reference mapped the highest (94.94%, Table 2) among the four references. CSSS (55.34%, Table 3) and W7984 (85.61%, Table 4) were in between. Among the three wheat genomes, B genome had the most mapped SNPs and D genome the least across the four references, thus B chromosomes had much higher marker density than that in the D chromosomes. Among the four reference genomes, IWGSC RefSeq v1.0 reference provided the highest SNP density in almost all chromosomes. Among 21 chromosomes, chromosomes 2B and 3B had the highest SNP density, and chromosome 4D had the lowest (Tables 1–4).

**Table 1:** Summary of single nucleotide polymorphism types identified in three wheat genomes using the barley genome reference.

Allele	1A	2A	3A	4A	5A	6A	7A	A genome	1B	2B	3B	4B	5B	6B	7B	B genome	1D	2D	3D	4D	5D	6D	7D	D genome	NA	Total
Chromosome_size(Mbp)	798	899	828	856	827	705	814	5,727	849	928	993	821	870	913	900	6,274	605	729	771	649	750	713	728	4,945	-	16,946
No. of SNP	803	1,211	574	316	410	874	977	5,165	910	1,750	1,537	471	1,277	689	613	7,247	414	709	160	57	330	470	620	2,760	50,388	65,560
Density (SNP/Mbp)	1.01	1.35	0.69	0.37	0.50	1.24	1.20	0.90	1.07	1.89	1.55	0.57	1.47	0.75	0.68	1.16	0.68	0.97	0.21	0.09	0.44	0.66	0.85	0.56	-	3.87
Transition	537	812	378	196	280	603	639	3,445	623	1,161	1,035	298	836	454	417	4,824	247	444	98	43	200	288	394	1,714	35,012	44,995
A/G	257	413	160	83	135	287	307	1,642	300	512	477	131	390	215	196	2,221	108	213	42	24	86	132	193	798	16,495	21,156
C/T	234	322	182	86	126	242	269	1,461	261	491	419	134	363	191	173	2,032	104	172	43	16	87	128	161	711	14,387	18,591
T/C	23	39	14	17	9	34	38	174	29	68	67	15	31	29	26	265	16	32	6	2	14	13	21	104	1,957	2,500
G/A	23	38	22	10	10	40	25	168	33	90	72	18	52	19	22	306	19	27	7	1	13	15	19	101	2,173	2,748
Transversion	266	399	196	120	130	271	338	1,720	287	589	502	173	441	235	196	2,423	167	265	62	14	130	182	226	1,046	15,376	20,565
A/T	37	69	26	20	17	43	41	253	35	77	81	32	62	47	22	356	29	38	12	3	16	21	37	156	2,137	2,902
A/C	52	113	42	30	26	58	75	396	60	139	119	40	118	53	45	574	46	56	16	1	32	41	50	242	3,597	4,809
T/A	3	8	6	4	4	5	15	45	6	11	16	2	3	8	5	51	3	3	1	0	1	1	2	11	314	421
T/G	3	7	2	1	2	5	8	28	9	25	18	6	7	4	8	77	5	6	2	1	2	7	3	26	453	584
C/A	8	10	5	2	5	4	9	43	9	10	11	2	9	1	8	50	3	11	3	0	2	6	3	28	437	558
C/G	88	115	71	41	37	97	102	551	101	194	131	44	152	65	69	756	48	87	12	5	46	61	67	326	4,665	6,298
G/T	64	69	36	18	31	44	70	332	54	108	108	41	75	46	33	465	25	54	13	3	24	32	52	203	3,140	4,140
G/C	11	8	8	4	8	15	18	72	13	25	18	6	15	11	6	94	8	10	3	1	7	13	12	54	633	853
Ts %	66.87	67.05	65.85	62.03	68.29	68.99	65.40	66.70	68.46	66.34	67.34	63.27	65.47	65.89	68.03	66.57	59.66	62.62	61.25	75.44	60.61	61.28	63.55	62.10	69.48	68.63
Tv %	33.13	32.95	34.15	37.97	31.71	31.01	34.60	33.30	31.54	33.66	32.66	36.73	34.53	34.11	31.97	33.43	40.34	37.38	38.75	24.56	39.39	38.72	36.45	37.90	30.52	31.37
Ts/Tv ratio	2.02	2.04	1.93	1.63	2.15	2.23	1.89	2.00	2.17	1.97	2.06	1.72	1.90	1.93	2.13	1.99	1.48	1.68	1.58	3.07	1.54	1.58	1.74	1.64	2.28	2.19

**Table 2:** Summary of single nucleotide polymorphism types identified in three wheat genomes using the wheat the IWGSC RefSeq v1.0.

Allele	1A	2A	3A	4A	5A	6A	7A	A genome	1B	2B	3B	4B	5B	6B	7B	B genome	1D	2D	3D	4D	5D	6D	7D	D genome	NA
No. of SNP	3,399	4,001	2943	3,684	2,850	2,683	4,556	24,116	4,424	5,083	5,350	2,055	4,054	4,776	4,055	29,797	1,370	2,029	1,162	466	798	993	1,510	8,328	3,319
Density (SNP/Mbp)	4.26	4.45	3.55	4.3	3.45	3.81	5.6	4.21	5.21	5.48	5.39	2.5	4.66	5.23	4.51	4.75	2.26	2.78	1.51	0.72	1.06	1.39	2.07	1.68	-
Transition	2,390	2,873	2,023	2,523	1,946	1,833	3,183	16,771	3,072	3,502	3,729	1,389	2,774	3,337	2,872	20,675	863	1,310	754	321	537	623	958	5,366	2,183
A/G	1,121	1,397	975	1,185	886	847	1,452	7,863	1,482	1,643	1,721	650	1,273	1,587	1,344	9,700	412	604	340	160	251	305	466	2,538	1,055
C/T	1,004	1,172	812	1,051	855	766	1,347	7,007	1,263	1,384	1,529	596	1,190	1,380	1,196	8,538	353	526	315	129	217	253	392	2,185	861
T/C	131	157	112	130	85	105	186	906	145	221	215	67	144	181	162	1,135	45	94	48	12	31	32	55	317	142
G/A	134	147	124	157	120	115	198	995	182	254	264	76	167	189	170	1,302	53	86	51	20	38	33	45	326	125
Transversion	1,009	1,128	920	1,161	904	850	1,373	7,345	1,352	1,581	1,621	666	1,280	1,439	1,183	9,122	507	719	408	145	261	370	552	2,962	1,136
A/T	140	181	126	180	116	109	184	1,036	195	234	236	96	162	207	145	1,275	69	98	56	21	45	54	97	440	151
A/C	219	296	209	263	207	204	341	1,739	307	373	390	154	306	357	285	2,172	116	160	94	28	61	89	115	663	235
T/A	18	28	23	22	19	10	28	148	27	36	41	13	25	29	23	194	9	12	11	2	4	6	4	48	31
T/G	17	25	20	29	36	20	33	180	33	56	59	20	33	40	45	286	15	22	14	2	6	12	12	83	35
C/A	24	31	21	28	25	29	38	196	36	41	43	19	44	28	43	254	6	23	9	4	9	12	15	78	30
C/G	314	319	297	362	262	274	397	2,225	429	468	456	187	390	440	385	2,755	160	234	125	45	68	103	180	915	403
G/T	243	213	183	212	207	167	302	1,527	264	308	335	154	267	276	218	1,822	104	134	83	36	59	74	101	591	200
G/C	34	35	41	65	32	37	50	294	61	65	61	23	53	62	39	364	28	36	16	7	9	20	28	144	51
Ts %	70.31	71.81	68.74	68.49	68.28	68.32	69.86	69.54	69.44	68.90	69.70	67.59	68.43	69.87	70.83	69.39	62.99	64.56	64.89	68.88	67.29	62.74	63.44	64.43	65.77
Tv %	29.69	28.19	31.26	31.51	31.72	31.68	30.14	30.46	30.56	31.10	30.30	32.41	31.57	30.13	29.17	30.61	37.01	35.44	35.11	31.12	32.71	37.26	36.56	35.57	34.23
Ts/Tv ratio	2.37	2.55	2.20	2.17	2.15	2.16	2.32	2.28	2.27	2.22	2.30	2.09	2.17	2.32	2.43	2.27	1.70	1.82	1.85	2.21	2.06	1.68	1.74	1.81	1.92

**Table 3:** Summary of single nucleotide polymorphism types identified in three wheat genomes using the wheat Chinese Spring survey sequence (CSSS) assembly.

Allele	1A	2A	3A	4A	5A	6A	7A	A genome	1B	2B	3B	4B	5B	6B	7B	B genome	1D	2D	3D	4D	5D	6D	7D	D genome	NA
No. of SNP	2,151	2,578	1,188	1,647	898	1,926	2,057	12,445	2,464	3,782	4,307	1,519	3,408	1,661	1,517	18,658	906	1,019	348	114	657	911	1,220	5,175	29,282
Density (SNP/Mbp)	2.7	2.87	1.43	1.92	1.09	2.73	2.53	2.17	2.9	4.08	4.34	1.85	3.92	1.82	1.69	2.97	1.5	1.4	0.45	0.18	0.88	1.28	1.68	1.05	-
Transition	1,490	1,830	813	1,113	606	1,326	1,417	8,595	1,707	2,671	2,994	1,026	2,372	1,116	1,049	12,935	568	647	216	92	440	574	796	3,333	20,132
A/G	704	897	371	489	273	591	645	3,970	793	1,214	1,394	472	1,114	527	474	5,988	263	293	88	53	210	262	379	1,548	9,650
C/T	632	732	346	478	277	577	619	3,661	722	1,117	1,219	449	995	472	462	5,436	235	271	93	33	183	251	326	1,392	8,102
T/C	74	104	46	68	19	69	75	455	89	153	169	56	116	59	65	707	36	45	13	4	25	29	46	198	1,140
G/A	80	97	50	78	37	89	78	509	103	187	212	49	147	58	48	804	34	38	22	2	22	32	45	195	1,240
Transversion	661	748	375	534	292	600	640	3,850	757	1,111	1,313	493	1,036	545	468	5,723	338	372	132	22	217	337	424	1,842	9,150
A/T	87	136	50	71	39	90	91	564	106	145	195	76	125	93	58	798	45	57	19	4	33	50	61	269	1,271
A/C	146	199	83	130	56	125	159	898	155	267	313	96	253	131	120	1,335	77	83	33	4	51	77	106	431	2,145
T/A	9	17	9	8	13	8	17	81	15	18	29	10	17	21	9	119	7	7	1	0	1	3	4	23	198
T/G	8	13	8	8	12	17	17	83	20	36	46	19	28	9	18	176	13	9	4	1	6	14	8	55	270
C/A	17	22	8	11	7	21	15	101	21	39	32	10	40	6	16	164	8	12	3	0	8	11	9	51	242
C/G	203	197	127	174	91	193	181	1,166	240	335	368	147	316	151	156	1,713	106	121	38	8	66	98	138	575	2,844
G/T	167	142	76	103	62	126	138	814	165	238	282	118	215	105	74	1,197	68	68	24	5	43	63	80	351	1,778
G/C	24	22	14	29	12	20	22	143	35	33	48	17	42	29	17	221	14	15	10	0	9	21	18	87	402
Ts %	69.27	70.99	68.43	67.58	67.48	68.85	68.89	69.06	69.28	70.62	69.51	67.54	69.60	67.19	69.15	69.33	62.69	63.49	62.07	80.70	66.97	63.01	65.25	64.41	68.75
Tv %	30.73	29.01	31.57	32.42	32.52	31.15	31.11	30.94	30.72	29.38	30.49	32.46	30.40	32.81	30.85	30.67	37.31	36.51	37.93	19.30	33.03	36.99	34.75	35.59	31.25
Ts/Tv ratio	2.25	2.45	2.17	2.08	2.08	2.21	2.21	2.23	2.25	2.40	2.28	2.08	2.29	2.05	2.24	2.26	1.68	1.74	1.64	4.18	2.03	1.70	1.88	1.81	2.20

**Table 4:** Summary of single nucleotide polymorphism types identified in three wheat genomes using the wheat W7984 assembly.

Allele	1A	2A	3A	4A	5A	6A	7A	A genome	1B	2B	3B	4B	5B	6B	7B	B genome	1D	2D	3D	4D	5D	6D	7D	D genome	NA
No. of SNP	2,907	3,434	2,645	3,299	1,921	2,455	4,023	20,684	3,813	4,649	5,067	1,986	3,686	4,455	3,792	27,448	1,232	1,804	1,128	428	897	1,039	1,465	7,993	9,435
Density (SNP/Mbp)	3.64	3.82	3.19	3.85	2.32	3.48	4.94	3.61	4.49	5.01	5.10	2.42	4.24	4.88	4.21	4.37	2.04	2.47	1.46	0.66	1.20	1.46	2.01	1.62	-
Transition	2,061	2,454	1,819	2,291	1,296	1,689	2,803	14,413	2,615	3,172	3,541	1,348	2,521	3,103	2,671	18,971	776	1,149	717	296	588	662	934	5,122	6,489
A/G	957	1,190	858	1,053	597	787	1,294	6,736	1,250	1,465	1,635	609	1,160	1,468	1,240	8,827	368	518	325	160	276	330	434	2,411	3,182
C/T	881	1,000	741	975	570	703	1,184	6,054	1,081	1,274	1,455	591	1,083	1,287	1,123	7,894	322	469	302	108	243	270	395	2,109	2,534
T/C	108	139	103	112	46	92	158	758	136	205	203	75	126	165	149	1,059	32	86	46	16	29	34	53	296	387
G/A	115	125	117	151	83	107	167	865	148	228	248	73	152	183	159	1,191	54	76	44	12	40	28	52	306	386
Transversion	846	980	826	1,008	625	766	1,220	6,271	1,198	1,477	1,526	638	1,165	1,352	1,121	8,477	456	655	411	132	309	377	531	2,871	2,946
A/T	99	170	112	149	83	98	165	876	174	199	226	84	159	188	135	1,165	67	88	53	17	48	59	90	422	439
A/C	189	252	193	229	130	181	299	1,473	273	356	357	142	274	337	272	2,011	107	145	98	28	69	86	99	632	693
T/A	12	22	26	14	19	12	19	124	24	31	37	16	23	26	24	181	9	12	11	1	3	5	8	49	67
T/G	18	19	18	28	28	23	22	156	32	49	56	21	32	36	43	269	10	21	12	5	7	10	14	79	80
C/A	19	28	21	24	14	24	32	162	33	37	42	20	39	26	43	240	9	23	5	3	11	12	13	76	80
C/G	276	273	247	315	186	248	371	1,916	377	447	426	184	355	414	358	2,561	143	215	130	34	91	109	171	893	928
G/T	211	186	171	196	144	151	265	1,324	232	299	325	149	239	270	199	1,713	89	120	83	35	66	71	109	573	530
G/C	22	30	38	53	21	29	47	240	53	59	57	22	44	55	47	337	22	31	19	9	14	25	27	147	129
Ts %	70.90	71.46	68.77	69.45	67.46	68.80	69.67	69.68	68.58	68.23	69.88	67.88	68.39	69.65	70.44	69.12	62.99	63.69	63.56	69.16	65.55	63.72	63.75	64.08	68.78
Tv %	29.10	28.54	31.23	30.55	32.54	31.20	30.33	30.32	31.42	31.77	30.12	32.12	31.61	30.35	29.56	30.88	37.01	36.31	36.44	30.84	34.45	36.28	36.25	35.92	31.22
Ts/Tv ratio	2.44	2.50	2.20	2.27	2.07	2.20	2.30	2.30	2.18	2.15	2.32	2.11	2.16	2.30	2.38	2.24	1.70	1.75	1.74	2.24	1.90	1.76	1.76	1.78	2.20

Transitions were the most observed nucleotide variations (68.63%) including A/G (32.27%), C/T (28.36%), C/G (9.61%), A/C (7.34%), G/T (6.31%) and A/T (4.43%) transition types (Table 1). Using the barley reference genome, more transition-type SNPs were identified in A (3,445) and B (4,824) genomes than that in the D genome (1,714). The transition/transversion (Ts/Tv) SNP ratios from the A and B genomes (2.0) were relatively higher than that (1.64) from the D genome (Table 1). A similar trend in SNP types was observed for the CSSS assembly, but its transition/transversion SNP ratios were higher than those from the barley reference genome with 2.23 for the A genome, 2.26 for the B genome and 1.81 for the D genome (Table 3). The W7984 assembly and IWGSC RefSeq v1.0 had similar transition/transversion ratios to the CSSS assembly, but with slightly higher numbers of total SNPs (Tables 2 and 4).

**Fig 2:**
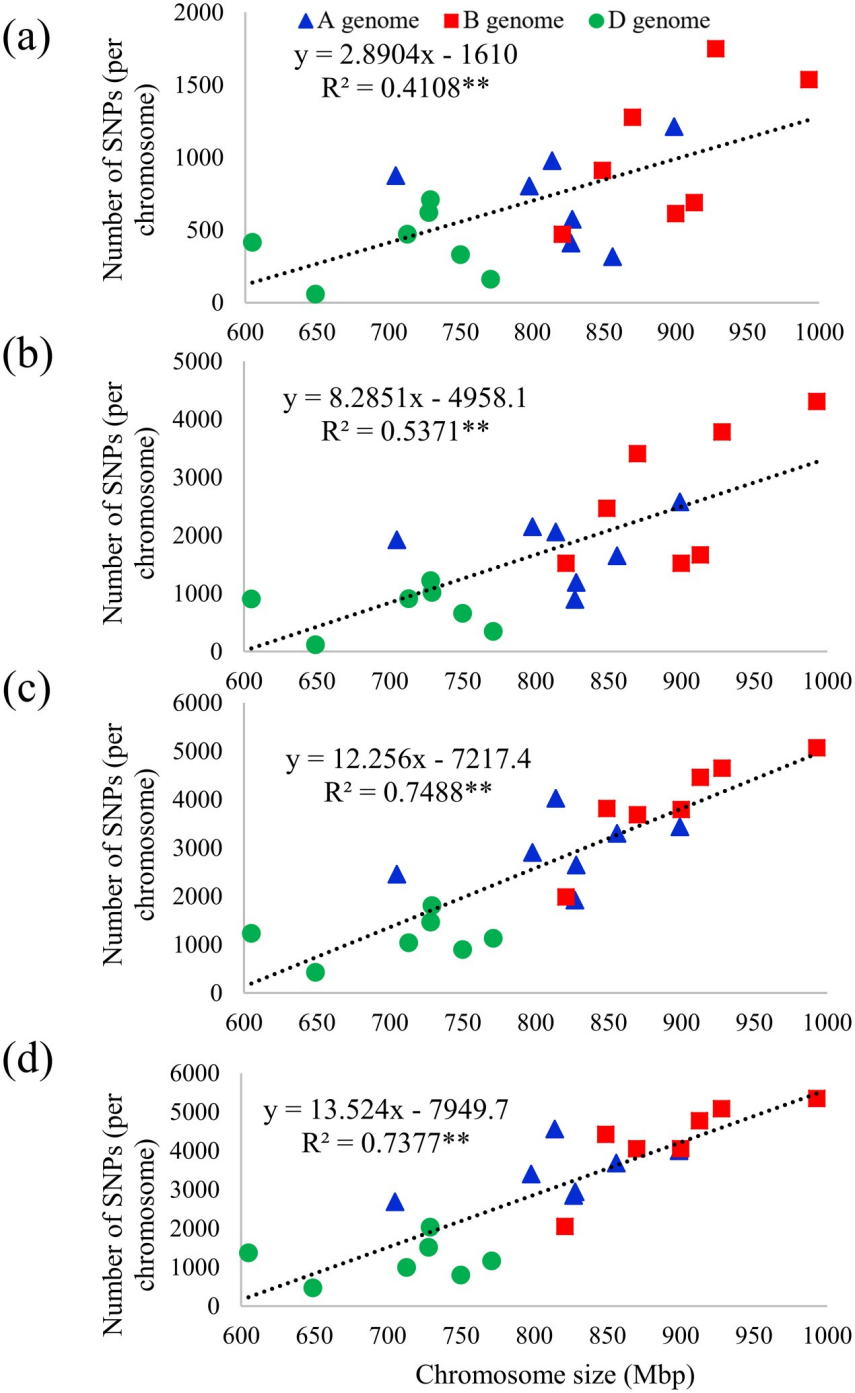
Relationship between the number of SNPs per chromosome and chromosome sizes when (a) barley genome, (b) Chinese Spring survey sequence (CSSS), (c) W7984, and (d) IWGSC RefSeq v1.0 reference was used to call SNPs.

The numbers of SNPs per chromosome were significantly correlated to the chromosome sizes (Mbp) in all four references. Although they were all significant, the correlations were much lower for the barley reference genome (R^2^ ~ 0.41**, Fig 2a) and wheat CSSS assembly (R^2^ ~ 0.54**, Fig 2b) than those for W7984 assembly (R^2^ ~ 0.75**, Fig 2c) and IWGSC RefSeq v1.0 (R^2^ ~ 0.74**, Fig 2d).

Five sequencing runs (three runs of the library 1 and two runs of the library 2) generated 355,197,375 total reads and 5,571 SNPs with less than 20% missing data. The number of SNPs was almost doubled (10,213) after adding one additional sequencing run of library 1 (total six runs) and tripled (16,506) after adding three sequencing runs of library 1 (total eight runs). For the number of SNPs with <80% missing data, increasing number of sequencing runs from five (45,624 SNPs) to eight (65,560 SNPs) only increased 44% SNPs. However, imputation reduced much more missing data than increasing sequencing runs from five to eight although imputation efficiency is different among the four references (Table 5). The numbers of SNPs with <20% missing data increased only three times when sequencing runs were increased from five to eight (Table 5), but increased 2.8 times (the barley reference genome), 5.1 times (CSSS), 7.0 times (W7984) and 7.8 times (IWGSC RefSeq v1.0) after imputation from the data of five runs. Imputation using IWGSC RefSeq v1.0 reference generated the most SNPs with only 4.9% missing data in the final imputed dataset; the barley reference genome imputed the least SNPs with 65.5% missing data after imputation; and wheat CSSS and W7984 were in between with 38.0% and 12.5% missing data after imputation (Table 5). These results indicate that although both imputation and increasing sequencing depth can quickly fill up missing data, imputation can reduce more missing data and therefore detect more SNPs than increasing sequencing depth with the highest increase for SNPs with <20% missing data.

**Table 5:** Numbers of SNPs called after five (3 runs of the library 1 and 2 runs of the library 2), six (4 runs of the library 1 and 2 runs of the library 2), seven (5 runs of the library 1 and 2 runs of the library 2) and eight (6 runs of the library 1 and 2 runs of the library 2) sequencing runs of the two GBS libraries with and without imputation using barley genome and Chinese Spring survey sequence (CSSS) and W7984 assembly and Wheat IWGSC RefSeq v1.0 reference.

Imputation methods	Missing data	Number of SNPs											
		Five sequencing runs (355,197,375 reads)			Six sequencing runs (419,373,662 reads)			Seven sequencing runs (486,646,481 reads)			Eight sequencing runs (566,439,207 reads)		
		<20%	<50%	<80%	<20%	<50%	<80%	<20%	<50%	<80%	<20%	<50%	<80%
Without imputation		5,571	26,773	45,624	10,213	31,472	53,241	13,746	35,380	59,766	16,506	38,642	65,560
Barley		15,738	30,442	45,624	20,142	35,320	53,241	23,526	39,269	59,766	26,284	42,706	65,560
Wheat CSSS		28,285	36,811	45,624	34,135	42,782	53,241	38,783	47,831	59,766	42,877	52,357	65,560
Wheat W7984		39,906	42,532	45,624	46,819	49,634	53,241	52,529	55,605	59,766	57,703	60,900	65,560
Wheat IWGSC RefSeq v1.0		43,365	44,410	45,624	50,735	51,924	53,241	57,065	58,361	59,766	62,740	64,083	65,560

Imputation accuracy was calculated for each reference by comparing the SNP data imputed from five, six or seven sequencing runs to the real SNP data from eight runs (Table 6). All four references provided high imputation accuracy. Among them, the IWGSC RefSeq v1.0 provided the lowest imputation accuracy (84.16%) although it imputed the most data points in all run combinations. The other three references had relatively higher accuracies from 87.31% for CSSS reference to 89.80% for W7984 reference (Table 6). The number of imputed data points was the highest using five sequencing runs, and the lowest using the data from eight sequencing runs. For all references and sequencing runs, even though the imputed data points per chromosome were much lower in the D genome than those in the A and B genomes in general, the D genome had the highest imputation accuracy and the B genome the lowest (Table 6).

**Table 6:** Numbers of data points per chromosome after imputation with barley genome, Chinese Spring survey sequence (CSSS), W7984 and IWGSC RefSeq v1.0 references and imputation accuracy calculated by comparing imputed data of five, six and seven runs with actual SNP data generated from eight sequencing runs.

Run	Reference	Missing data points	A genome	B genome	D genome	Total
Five runs	Barley	Total data points	157,689	221,204	85,281	464,174
		Correctly imputed	141,111	195,450	77,230	413,791
		Accuracy	89.49	88.36	90.56	89.15
	CSSS	Total data points	374,452	448,904	125,864	949,220
		Correctly imputed	339,919	401,393	114,507	855,819
		Accuracy	90.78	89.42	90.98	90.16
	W7984	Total data points	556,620	637,680	186,281	1,380,581
		Correctly imputed	502,417	568,400	169,075	1,239,892
		Accuracy	90.26	89.14	90.76	89.81
	IWGSC RefSeq v1.0	Total data points	691,863	841,893	243,017	1,776,773
		Correctly imputed	584,917	694,667	209,385	1,488,969
		Accuracy	84.54	82.51	86.16	83.80
Six runs	Barley	Total data points	23,138	22,979	11,552	57,669
		Correctly imputed	20,356	20,016	10,294	50,666
		Accuracy	87.98	87.11	89.11	87.86
	CSSS	Total data points	74,520	103,888	29,095	207,503
		Correctly imputed	63,312	86,484	25,494	175,290
		Accuracy	84.96	83.25	87.62	84.48
	W7984	Total data points	393,307	517,916	154,857	1,066,080
		Correctly imputed	363,152	473,970	143,550	980,672
		Accuracy	92.33	91.51	92.70	91.99
	IWGSC RefSeq v1.0	Total data points	467,224	574,541	162,471	1,204,236
		Correctly imputed	397,638	475,980	140,790	1,014,408
		Accuracy	85.11	82.85	86.66	84.24
Seven runs	Barley	Total data points	25,242	33,810	12,195	71,247
		Correctly imputed	22,637	29,634	11,207	63,478
		Accuracy	89.68	87.65	91.90	89.10
	CSSS	Total data points	67,293	104,296	25,254	196,843
		Correctly imputed	59,247	89,933	22,647	171,827
		Accuracy	88.04	86.23	89.68	87.29
	W7984	Total data points	154,672	214,222	59,594	428,488
		Correctly imputed	135,735	186,183	53,429	375,347
		Accuracy	87.76	86.91	89.65	87.60
	IWGSC RefSeq v1.0	Total data points	233,321	289,654	80,048	603,023
		Correctly imputed	199,037	240,517	69,725	509,279
		Accuracy	85.31	83.04	87.10	84.45

**Fig 3:**
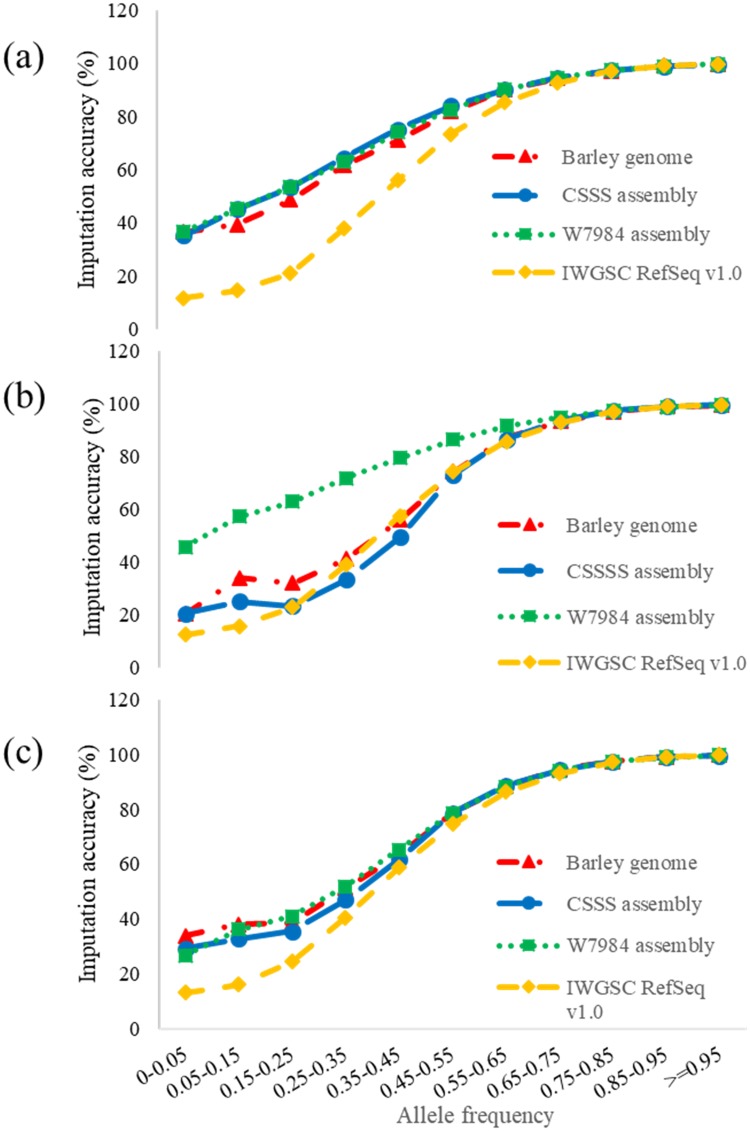
Relationship between imputation accuracy and allele frequency for (a) five, (b) six, (c) seven runs imputed with eight runs using barley genome, Chinese Spring survey sequence (CSSS), W7984 and IWGSC RefSeq 1.0 in Iranian wheat GBS data.

**Fig 4:**
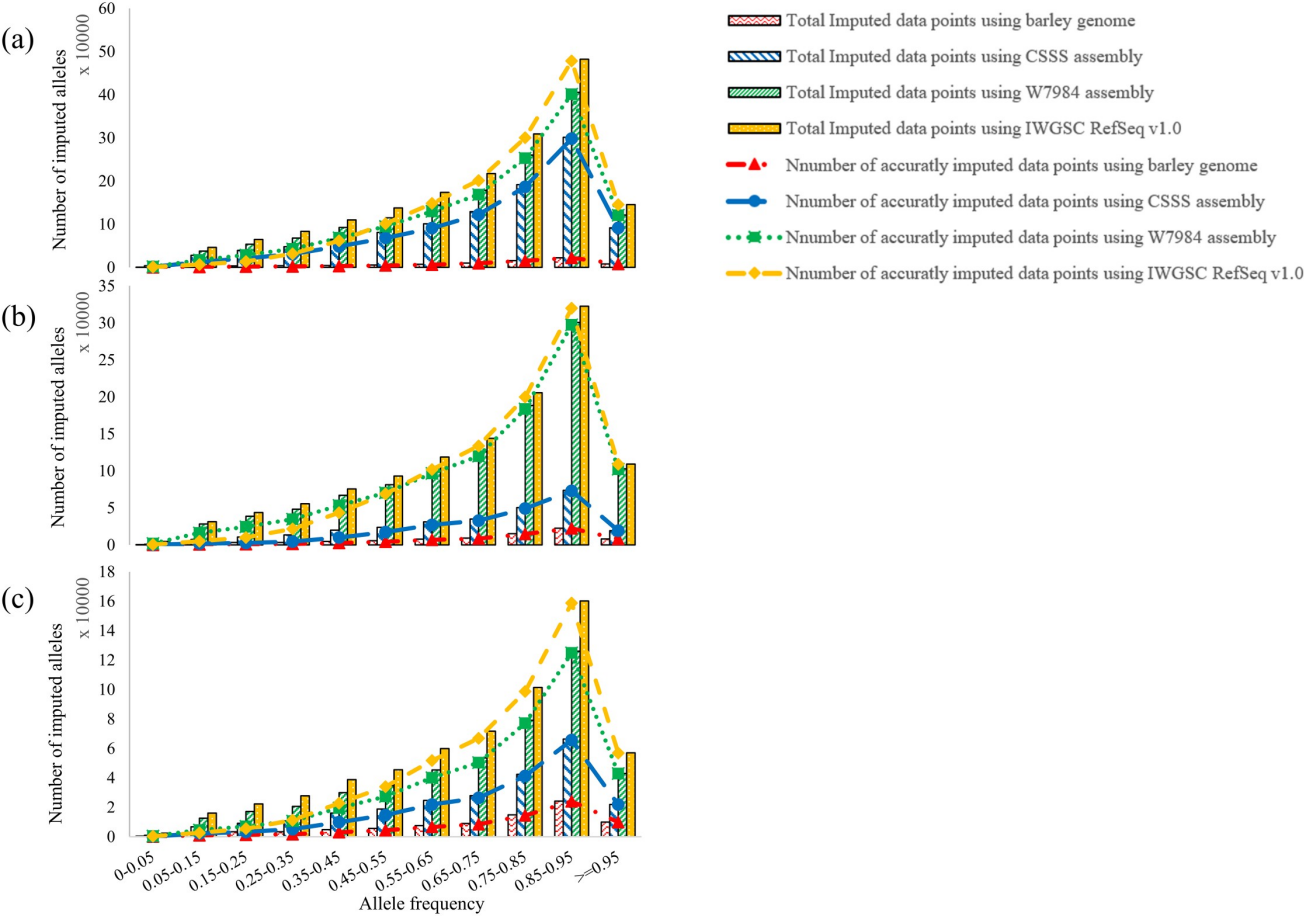
Number of total and correctly imputed alleles for different allele frequencies for (a) five, (b) six, (c) seven runs imputed with eight runs using barley genome, Chinese Spring survey sequence (CSSS), W7984 and IWGSC RefSeq 1.0 in Iranian wheat GBS data.

The imputation accuracy increased with the increase in allele frequency from 25.7% accuracy for allele frequency < 5% and 99.7% accuracy for allele frequency > 95% (Fig 3). The positive correlations were observed for all four references. The imputation accuracy reached 94% when allele frequency was >65%. Relatively lower imputation accuracy of IWGSC RefSeq v1.0 than other references mainly occurred at those alleles with frequency <35% (Fig 3b and 3c) where W7984 assembly provided more accurate imputation than other references. About 75% of imputed SNPs were distributed in allele frequencies between 0.55 and 0.95 (Fig 4), and had high mean imputation accuracies from 88.0% to 99.7%. The difference in imputation accuracy in this allele frequency range was negligible among the four references (Fig 4).

## Discussion

Advancements in next-generation sequencing technology and high-throughput SNP genotyping can greatly accelerate crop breeding process if properly deployed [37]. GBS technology not only significantly improves throughput, but also greatly reduces SNP genotyping costs by reducing genome complexity and multiplexing samples [13]. Although GBS can generate a large number of SNP markers, its application in association mapping and genomics-assisted breeding can be limited by massive amount of missing data when low coverage sequencing is conducted [4, 38]. Biologically, missing SNP calls in GBS datasets can be due to presence-absence variation and/or differential methylation in restriction sites. Technically, genome complexity, low library quality, and sequence coverage [27] are among the major contributors. Library complexity can be reduced by digesting sample DNA with restriction enzymes such as *PstI* and *MspI*. A combination of *PstI* and *MspI* enzymes has been successfully used for high quality wheat library construction [13]. The sequencing coverage is a function of genome complexity, multiplexing level, and output of a NGS platform [39]. In the current study, we constructed two libraries, library 1 with three plates of samples and library 2 with one plate of samples. The library 1 had six sequencing runs and the library 2 had two, thus each sample in both libraries had the same sequence depth. However, the samples in the library 1 produced more SNPs (14,781, 32,926 and 49,717 SNPs at <20%, <50%, and <80% missing data, respectively) than those from the library 2 (10,630, 23,931 and 26,198 SNPs at <20%, <50%, and <80% missing data, respectively), suggesting that raising multiplexing level and sequencing multiple times can significantly increase SNP number and reduce missing data in comparison with a library at lower multiplex level with the same sequencing depth. The results in this study showed that increasing number of SNPs with <20% missing data was concave up by increasing run number (Fig 1a) while increasing numbers of SNPs with <50% and <80% missing data were concave down (Fig 1b and 1c), suggesting increasing run number can quickly reduce missing data, but slowly increase in total numbers of SNPs.

Typically, two strategies can be used to reduce missing data: increasing sequencing depth or imputing missing data using a reference genome. Increasing sequence depth can be achieved through lowering multiplexing level in a fixed run number or increasing sequencing runs of a highly multiplexed library. Both methods will result in an increase in per-sample cost. As indicated previously, lowering multiplex level may not increase SNP number as expected. Thus, increasing number of sequencing runs can be an option. In this study, the first library was run six times and the number of SNPs was significantly increased especially for the number of SNPs with <20% missing data (Fig 1), indicating that increasing number of runs significantly decreased the number of missing data and therefore increased number of usable SNPs. However, this also increased the per-sample cost significantly. Imputing missing data is an effective approach to minimize missing data without increasing sequencing costs. Imputed data can be very accurate if a high-quality genome reference is available and SNP markers can be accurately aligned on the physical map [27]. However, in the case that genome reference is absent or incomplete, such imputation is challenging. In this study, we compared imputation efficiency and accuracy among four genome references including the barley reference genome and three wheat references (CSSS, W7984 and IWGSC RefSeq v1.0) and found that all the references are useful for ordering GBS-SNPs and can significantly reduce missing data points and provide accurate imputation to leverage the application GBS markers in wheat [4] although imputation efficiency varied with completeness of genome references.

Before imputation, we were able to bioinformatically map 15,172 (~23.14% of total SNPs called), 36,278 (~55.34%), 56,125 (~85.61%) and 62,241 (94.94%) SNPs out of 65,560 SNPs with <80% missing data to the barley reference genome (Table 1), CSSS (Table 2), W7984 (Table 3) and IWGSC RefSeq v1.0 (Table 4) references, respectively. That the most SNPs were mapped to IWGSC RefSeq v1.0 among the four reference genomes may be due to that the newest reference has the best genome coverage. For all four references, the highest number of SNPs were mapped to the B genome with 47.77%, 51.43%, 48.91% and 47.87% of totally mapped SNPs to the barley reference genome, CSSS, W7984 and IWGSC RefSeq v1.0 references, respectively, and the lowest number of SNPs were mapped to the D genome with 18.19%, 14.26%, 14.24% and 13.38% of totally mapped SNPs, respectively (Tables 1, 2, 3 and 4). The number of SNPs mapped to the A and B genomes were 1.8 and 3.6 times higher than those mapped on the D genome, whereas the differences in the numbers of mapped SNPs between the D genome and the A and B genomes from previous reports were even higher (about two-fold higher) than observed in this study [40–42], reflecting the most recent polyploidy bottleneck of hexaploid wheat [2, 43]. During the evolution of modern bread wheat, there has been extensive gene flow between hexaploid *T*. *aestivum* and tetraploid emmer wheat (AABB), while gene flow between the hexaploid and *Ae*. *tauschii* (DD) might have not occurred [44–47], which might explain higher polymorphism on the A and B genomes than on the D genome [31, 48]. The greatest number of SNPs was mapped to the chromosome 3B and the least number of SNPs was mapped to the chromosome 4D, which agrees with Edae et al. [4] using W7984 and CSSS assemblies.

The number of transition-type SNPs (68.63%) with majority of A/G (32.27%) and C/T (28.36%) transition was much higher than transversion-type SNPs with an average Ts/Tv SNP ratio of 2.19 (Table 1), which agrees with several previous studies on hexaploid wheat [31, 49–53] and barley [54–56] where Ts/Tv SNP ratios was from 1.59 to 2.12. A/G and C/T types of mutations are usually due to methylation of cytosine that can be easily achieved from spontaneous deamination and transition to a thymine [57]. In this study, the Ts/Tv SNP ratios from the A and B genomes were significantly higher than that from the D genome, which most likely due to high methylation occurred in the A and B genomes during the two rounds of polyploidization [49], whereas D genome has been through only one round of such polyploidization during the hexaploid wheat evolution [58].

Among the four reference genomes, IWGSC RefSeq v1.0 has the best wheat genome sequence coverage and assembly quality therefore it is expected that the IWGSC RefSeq v1.0 generated the most imputed data points (1,776,773) from the five sequencing runs and the barley reference genome (464,174) generated the least (Table 6). However, W7984 assembly had the highest imputation accuracy. An obvious relationship was not observed between imputation accuracy and chromosome size and between imputation accuracy and number of missing data per chromosome. The percentages of imputed SNPs were much higher in the A (~38.69%) and B (~48.03%) genomes than that in the D genome (~13.28). The numbers of imputed data points in the A and B chromosomes were much higher than that from the D chromosomes, but imputation accuracy of the SNPs in the D chromosomes was the highest (Table 6). This could be owing to the low polymorphism level that resulted in a relatively low number of imputed SNPs in the D genome [59]. A high LD level on the D chromosomes may also contribute to its higher imputation accuracy than that in the A and B genomes [43, 60–63] as observed in several other studies [36, 64–66].

A high positive correlation was observed between imputation accuracy and allele frequency. Based on different references and runs, the greatest number of imputed data points was observed in allele frequencies of 0.55 to 0.95 with imputation accuracy from 88 to 99% (Fig 4a, 4b and 4c). Although the numbers of imputed data points using IWGSC RefSeq v1.0 were higher than those using other three references in different allele frequencies, especially after six runs, the imputation accuracy using IWGSC RefSeq v1.0 was much lower than other references in the lower allele frequency. However, imputation accuracy for 75% of missing data were similar among four genome references, and only the missing data with lower allele frequencies showed difference in imputation accuracy among references. SNPs with a low minor allele frequency (MAF < 10%) was reported to have significantly lower power to detect true trait-marker association [67], thus, they were removed in many GWAS [68]. Since IWGSC RefSeq v1.0 imputed the most missing data points, it can be used to impute missing data if SNPs with MAF < 10% was removed in a study. However, in the cases where rare variants with MAF <10% might play a more important role than common SNPs with a MAF >10% [69], imputation using W7984 may improve imputation accuracy.

## Conclusions

Imputation using genome references is an effective tool to fill up massive missing genotypic data generated from GBS. Among the four references (the barley reference genome and wheat reference genomes of CSSS, W7984 and IWGSC RefSeq v1.0) used for imputation, IWGSC RefSeq v1.0 imputed the greatest number of missing data points with adequate imputation accuracy, especially for those alleles with high frequencies. For those alleles with low allele frequency, W7984 assembly showed the best imputation accuracy although imputed number of missing data points was slightly lower than the IWGSC RefSeq v1.0 reference. Therefore, they both can be used as reference genomes to impute missing GBS data in wheat breeding and genetic research.

## References

[1] FAO. High Level Expert Forum—How to Feed the World in 2050. Food and Agricultural Organization of the United Nations Rome; 2009.

[2] Marcussen T, Sandve SR, Heier L, Spannagl M, Pfeifer M, Jakobsen KS, et al Ancient hybridizations among the ancestral genomes of bread wheat. Science. 2014;345(6194):1250092 10.1126/science.1250092 25035499

[3] Ray DK, Mueller ND, West PC, Foley JA. Yield trends are insufficient to double global crop production by 2050. PloS One. 2013;8(6):e66428 10.1371/journal.pone.0066428 23840465 PMC3686737

[4] Edae EA, Bowden RL, Poland J. Application of Population Sequencing (POPSEQ) for Ordering and Imputing Genotyping-by-Sequencing Markers in Hexaploid Wheat. G3. 2015;5(12):2547–53. 10.1534/g3.115.020362 . 26530417 PMC4683627

[5] Elshire RJ, Glaubitz JC, Sun Q, Poland JA, Kawamoto K, Buckler ES, et al A robust, simple genotyping-by-sequencing (GBS) approach for high diversity species. PloS One. 2011;6(5):e19379 10.1371/journal.pone.0019379 . 21573248 PMC3087801

[6] He J, Zhao X, Laroche A, Lu ZX, Liu H, Li Z. Genotyping-by-sequencing (GBS), an ultimate marker-assisted selection (MAS) tool to accelerate plant breeding. Front Plant Sci. 2014;5:484 10.3389/fpls.2014.00484 . 25324846 PMC4179701

[7] Batley J, Edwards D. SNP applications in plants Association mapping in plants: Springer; 2007 p. 95–102.

[8] Kumar S, Banks TW, Cloutier S. SNP Discovery through Next-Generation Sequencing and Its Applications. Int J Plant Genomics. 2012;2012:831460 10.1155/2012/831460 . 23227038 PMC3512287

[9] Yan J, Kandianis CB, Harjes CE, Bai L, Kim E-H, Yang X, et al Rare genetic variation at Zea mays crtRB1 increases β-carotene in maize grain. Nature Genet. 2010;42(4):322 10.1038/ng.551 20305664

[10] Sato K, Nankaku N, Takeda K. A high-density transcript linkage map of barley derived from a single population. Heredity. 2009;103(2):110 10.1038/hdy.2009.57 19455180

[11] Bernardo A, Wang S, St Amand P, Bai G. Using Next Generation Sequencing for Multiplexed Trait-Linked Markers in Wheat. PloS One. 2015;10(12):e0143890 10.1371/journal.pone.0143890 . 26625271 PMC4666610

[12] Poland J, Endelman J, Dawson J, Rutkoski J, Wu S, Manes Y, et al Genomic Selection in Wheat Breeding using Genotyping-by-Sequencing. Plant Genome. 2012;5(3):103 10.3835/plantgenome2012.06.0006

[13] Poland JA, Brown PJ, Sorrells ME, Jannink J-L. Development of high-density genetic maps for barley and wheat using a novel two-enzyme genotyping-by-sequencing approach. PloS One. 2012;7(2):e32253 10.1371/journal.pone.0032253 22389690 PMC3289635

[14] Huang YF, Poland JA, Wight CP, Jackson EW, Tinker NA. Using genotyping-by-sequencing (GBS) for genomic discovery in cultivated oat. PloS One. 2014;9(7):e102448 10.1371/journal.pone.0102448 . 25047601 PMC4105502

[15] Meuwissen TH, Goddard ME. Accurate prediction of genetic values for complex traits by whole genome resequencing. Genetics. 2010. 10.1534/genetics.110.116590 PMC2881142 20308278

[16] Druet T, Macleod I, Hayes B. Toward genomic prediction from whole-genome sequence data: impact of sequencing design on genotype imputation and accuracy of predictions. Heredity. 2014;112(1):39 10.1038/hdy.2013.13 23549338 PMC3860159

[17] Marchini J, Howie B, Myers S, McVean G, Donnelly P. A new multipoint method for genome-wide association studies by imputation of genotypes. Nature Genet. 2007;39(7):906 10.1038/ng2088 17572673

[18] Li Y, Willer CJ, Ding J, Scheet P, Abecasis GR. MaCH: using sequence and genotype data to estimate haplotypes and unobserved genotypes. Genet Epidemiol. 2010;34(8):816–34. 10.1002/gepi.20533 21058334 PMC3175618

[19] Scheet P, Stephens M. A fast and flexible statistical model for large-scale population genotype data: applications to inferring missing genotypes and haplotypic phase. Am J Hum Genet. 2006;78(4):629–44. 10.1086/502802 16532393 PMC1424677

[20] Browning BL, Browning SR. A unified approach to genotype imputation and haplotype-phase inference for large data sets of trios and unrelated individuals. Am J Hum Genet. 2009;84(2):210–23. 10.1016/j.ajhg.2009.01.005 19200528 PMC2668004

[21] Whalen A, Gorjanc G, Ros-Freixedes R, Hickey JM. Assessment of the performance of different hidden Markov models for imputation in animal breeding. bioRxiv. 2017:227157. 10.1186/s12711-018-0416-8 PMC6142395 30223768

[22] Pei Y-F, Li J, Zhang L, Papasian CJ, Deng H-W. Analyses and comparison of accuracy of different genotype imputation methods. PloS One. 2008;3(10):e3551 10.1371/journal.pone.0003551 18958166 PMC2569208

[23] Verma S, Gupta S, Bandhiwal N, Kumar T, Bharadwaj C, Bhatia S. High-density linkage map construction and mapping of seed trait QTLs in chickpea (*Cicer arietinum* L.) using Genotyping-by-Sequencing (GBS). Sci Rep. 2015;5:17512 10.1038/srep17512 26631981 PMC4668357

[24] Chan AW, Hamblin MT, Jannink J-L. Evaluating imputation algorithms for low-depth genotyping-by-sequencing (GBS) data. PloS One. 2016;11(8):e0160733 10.1371/journal.pone.0160733 27537694 PMC4990193

[25] Hussain W, Baenziger PS, Belamkar V, Guttieri MJ, Venegas JP, Easterly A, et al Genotyping-by-sequencing derived high-density linkage map and its application to QTL mapping of flag leaf traits in bread wheat. Sci Rep. 2017;7(1):16394 10.1038/s41598-017-16006-z 29180623 PMC5703991

[26] Wang Y, Lin G, Li C, Stothard P. Genotype imputation methods and their effects on genomic predictions in cattle. Springer Science Reviews. 2016;4(2):79–98.

[27] Poland JA, Rife TW. Genotyping-by-Sequencing for Plant Breeding and Genetics. Plant Genome. 2012;5(3):92 10.3835/plantgenome2012.05.0005

[28] Mayer KF, Waugh R, Brown JW, Schulman A, Langridge P, Platzer M, et al A physical, genetic and functional sequence assembly of the barley genome. Nature. 2012;491(7426):711–6. 10.1038/nature11543 . 23075845

[29] International Wheat Genome Sequencing C. A chromosome-based draft sequence of the hexaploid bread wheat (*Triticum aestivum* L.) genome. Science. 2014;345(6194):1251788 10.1126/science.1251788 . 25035500

[30] Chapman JA, Mascher M, Buluc A, Barry K, Georganas E, Session A, et al A whole-genome shotgun approach for assembling and anchoring the hexaploid bread wheat genome. Genome Biol. 2015;16:26 10.1186/s13059-015-0582-8 . 25637298 PMC4373400

[31] Alipour H, Bihamta MR, Mohammadi V, Peyghambari SA, Bai G, Zhang G. Genotyping-by-Sequencing (GBS) Revealed Molecular Genetic Diversity of Iranian Wheat Landraces and Cultivars. Front Plant Sci. 2017;8:1293 10.3389/fpls.2017.01293 . 28912785 PMC5583605

[32] Saghai-Maroof MA, Soliman KM, Jorgensen RA, Allard R. Ribosomal DNA spacer-length polymorphisms in barley: Mendelian inheritance, chromosomal location, and population dynamics. Proc Natl Acad Sci. 1984;81(24):8014–8. 6096873 10.1073/pnas.81.24.8014 PMC392284

[33] Lu F, Lipka AE, Glaubitz J, Elshire R, Cherney JH, Casler MD, et al Switchgrass genomic diversity, ploidy, and evolution: novel insights from a network-based SNP discovery protocol. PLoS Genet. 2013;9(1):e1003215 10.1371/journal.pgen.1003215 23349638 PMC3547862

[34] Bradbury PJ, Zhang Z, Kroon DE, Casstevens TM, Ramdoss Y, Buckler ES. TASSEL: software for association mapping of complex traits in diverse samples. Bioinformatics. 2007;23(19):2633–5. 10.1093/bioinformatics/btm308 17586829

[35] Rabiner LR. A tutorial on hidden Markov models and selected applications in speech recognition. Proc IEEE. 1989;77(2):257–86.

[36] He S, Zhao Y, Mette MF, Bothe R, Ebmeyer E, Sharbel TF, et al Prospects and limits of marker imputation in quantitative genetic studies in European elite wheat (*Triticum aestivum* L.). BMC Genomics. 2015;16(1):168. 25886991 10.1186/s12864-015-1366-y PMC4364688

[37] Thomson MJ. High-Throughput SNP Genotyping to Accelerate Crop Improvement. Plant Breed Biotechnol. 2014;2(3):195–212. 10.9787/pbb.2014.2.3.195

[38] Davey JW, Hohenlohe PA, Etter PD, Boone JQ, Catchen JM, Blaxter ML. Genome-wide genetic marker discovery and genotyping using next-generation sequencing. Nature Rev Genet. 2011;12(7):499–510. 10.1038/nrg3012 . 21681211

[39] Andolfatto P, Davison D, Erezyilmaz D, Hu TT, Mast J, Sunayama-Morita T, et al Multiplexed shotgun genotyping for rapid and efficient genetic mapping. Genome Res. 2011;21(4):610–7. 10.1101/gr.115402.110 21233398 PMC3065708

[40] Allen AM, Barker GL, Berry ST, Coghill JA, Gwilliam R, Kirby S, et al Transcript‐specific, single‐nucleotide polymorphism discovery and linkage analysis in hexaploid bread wheat (*Triticum aestivum* L.). Plant Biotechnol J. 2011;9(9):1086–99. 10.1111/j.1467-7652.2011.00628.x 21627760

[41] Cavanagh CR, Chao S, Wang S, Huang BE, Stephen S, Kiani S, et al Genome-wide comparative diversity uncovers multiple targets of selection for improvement in hexaploid wheat landraces and cultivars. Proc Natl Acad Sci. 2013;110(20):8057–62. 10.1073/pnas.1217133110 . 23630259 PMC3657823

[42] Allen AM, Barker GL, Wilkinson P, Burridge A, Winfield M, Coghill J, et al Discovery and development of exome‐based, co‐dominant single nucleotide polymorphism markers in hexaploid wheat (*Triticum aestivum* L.). Plant Biotechnol J. 2013;11(3):279–95. 10.1111/pbi.12009 23279710

[43] Chao S, Dubcovsky J, Dvorak J, Luo M-C, Baenziger SP, Matnyazov R, et al Population-and genome-specific patterns of linkage disequilibrium and SNP variation in spring and winter wheat (*Triticum aestivum* L.). BMC Genomics. 2010;11(1):727. 21190581 10.1186/1471-2164-11-727 PMC3020227

[44] Berkman PJ, Visendi P, Lee HC, Stiller J, Manoli S, Lorenc MT, et al Dispersion and domestication shaped the genome of bread wheat. Plant Biotechnol J. 2013;11(5):564–71. 10.1111/pbi.12044 23346876

[45] Caldwell KS, Dvorak J, Lagudah ES, Akhunov E, Luo MC, Wolters P, et al Sequence polymorphism in polyploid wheat and their d-genome diploid ancestor. Genetics. 2004;167(2):941–7. 10.1534/genetics.103.016303 . 15238542 PMC1470897

[46] Dvorak J, Akhunov ED, Akhunov AR, Deal KR, Luo M-C. Molecular characterization of a diagnostic DNA marker for domesticated tetraploid wheat provides evidence for gene flow from wild tetraploid wheat to hexaploid wheat. Mol Biol Evol. 2006;23(7):1386–96. 10.1093/molbev/msl004 16675504

[47] Talbert L, Smith L, Blake N. More than one origin of hexaploid wheat is indicated by sequence comparison of low-copy DNA. Genome. 1998;41(3):402–7.

[48] Lai K, Lorenc MT, Lee HC, Berkman PJ, Bayer PE, Visendi P, et al Identification and characterization of more than 4 million intervarietal SNPs across the group 7 chromosomes of bread wheat. Plant Biotechnol J. 2015;13(1):97–104. 10.1111/pbi.12240 . 25147022

[49] Lorenc MT, Hayashi S, Stiller J, Lee H, Manoli S, Ruperao P, et al Discovery of Single Nucleotide Polymorphisms in Complex Genomes Using SGSautoSNP. Biology. 2012;1(2):370–82. 10.3390/biology1020370 . 24832230 PMC4009776

[50] Winfield MO, Wilkinson PA, Allen AM, Barker GL, Coghill JA, Burridge A, et al Targeted re‐sequencing of the allohexaploid wheat exome. Plant Biotechnol J. 2012;10(6):733–42. 10.1111/j.1467-7652.2012.00713.x 22703335

[51] Manickavelu A, Jighly A, Ban T. Molecular evaluation of orphan Afghan common wheat (*Triticum aestivum* L.) landraces collected by Dr. Kihara using single nucleotide polymorphic markers. BMC Plant Biol. 2014;14(1):320. 25432399 10.1186/s12870-014-0320-5 PMC4255927

[52] Cui F, Zhang N, Fan XL, Zhang W, Zhao CH, Yang LJ, et al Utilization of a Wheat660K SNP array-derived high-density genetic map for high-resolution mapping of a major QTL for kernel number. Sci Rep. 2017;7(1):3788 10.1038/s41598-017-04028-6 . 28630475 PMC5476560

[53] Rimbert H, Darrier B, Navarro J, Kitt J, Choulet F, Leveugle M, et al High throughput SNP discovery and genotyping in hexaploid wheat. PloS One. 2018;13(1):e0186329 10.1371/journal.pone.0186329 . 29293495 PMC5749704

[54] Turuspekov Y, Ormanbekova D, Rsaliev A, Abugalieva S. Genome-wide association study on stem rust resistance in Kazakh spring barley lines. BMC Plant Biol. 2016;16 Suppl 1:6 10.1186/s12870-015-0686-z . 26821649 PMC4895317

[55] Kono TJ, Fu F, Mohammadi M, Hoffman PJ, Liu C, Stupar RM, et al The Role of Deleterious Substitutions in Crop Genomes. Mol Biol Evol. 2016;33(9):2307–17. 10.1093/molbev/msw102 . 27301592 PMC4989107

[56] Bayer MM, Rapazote-Flores P, Ganal M, Hedley PE, Macaulay M, Plieske J, et al Development and Evaluation of a Barley 50k iSelect SNP Array. Front Plant Sci. 2017;8:1792 10.3389/fpls.2017.01792 . 29089957 PMC5651081

[57] Rosenberg MS, Subramanian S, Kumar S. Patterns of transitional mutation biases within and among mammalian genomes. Mol Biol Evol. 2003;20(6):988–93. 10.1093/molbev/msg113 12716982

[58] Lai K. Genome diversity in Triticum aestivum. PhD thesis, The University of Queensland, St Lucia, QLD. 2015.

[59] Jordan KW, Wang S, Lun Y, Gardiner LJ, MacLachlan R, Hucl P, et al A haplotype map of allohexaploid wheat reveals distinct patterns of selection on homoeologous genomes. Genome Biol. 2015;16:48 10.1186/s13059-015-0606-4 . 25886949 PMC4389885

[60] Akhunov ED, Akhunova AR, Anderson OD, Anderson JA, Blake N, Clegg MT, et al Nucleotide diversity maps reveal variation in diversity among wheat genomes and chromosomes. BMC Genomics. 2010;11(1):702. 21156062 10.1186/1471-2164-11-702 PMC3022916

[61] Chao S, Zhang W, Dubcovsky J, Sorrells M. Evaluation of genetic diversity and genome-wide linkage disequilibrium among US wheat (*Triticum aestivum* L.) germplasm representing different market classes. Crop Science. 2007;47(3):1018–30.

[62] Chen X, Min D, Yasir TA, Hu Y-G. Genetic diversity, population structure and linkage disequilibrium in elite Chinese winter wheat investigated with SSR markers. PloS One. 2012;7(9):e44510 10.1371/journal.pone.0044510 22957076 PMC3434133

[63] Wang S, Wong D, Forrest K, Allen A, Chao S, Huang BE, et al Characterization of polyploid wheat genomic diversity using a high-density 90,000 single nucleotide polymorphism array. Plant Biotechnol J. 2014;12(6):787–96. 10.1111/pbi.12183 . 24646323 PMC4265271

[64] Zhang Z, Druet T. Marker imputation with low-density marker panels in Dutch Holstein cattle. J Dairy Sci. 2010;93(11):5487–94. 10.3168/jds.2010-3501 20965364

[65] Rutkoski JE, Poland J, Jannink J-L, Sorrells ME. Imputation of unordered markers and the impact on genomic selection accuracy. G3. 2013;3(3):427–39. 10.1534/g3.112.005363 23449944 PMC3583451

[66] Torkamaneh D, Belzile F. Scanning and Filling: Ultra-Dense SNP Genotyping Combining Genotyping-By-Sequencing, SNP Array and Whole-Genome Resequencing Data. PloS One. 2015;10(7):e0131533 10.1371/journal.pone.0131533 . 26161900 PMC4498655

[67] Ardlie KG, Lunetta KL, Seielstad M. Testing for population subdivision and association in four case-control studies. Am J Hum Genet. 2002;71(2):304–11. 10.1086/341719 12096349 PMC379163

[68] Cupples LA, Arruda HT, Benjamin EJ, D’Agostino RB, Demissie S, DeStefano AL, et al The Framingham Heart Study 100K SNP genome-wide association study resource: overview of 17 phenotype working group reports. BioMed Central; 2007. 10.1186/1471-2350-8-S1-S1 PMC1995613 17903291

[69] McClellan J, King M-C. Genetic heterogeneity in human disease. Cell. 2010;141(2):210–7. 10.1016/j.cell.2010.03.032 20403315

